# Translation and psychometric evaluation of the Chinese version of the Charité Alarm Fatigue Questionnaire

**DOI:** 10.3389/fpsyg.2026.1723867

**Published:** 2026-02-04

**Authors:** Wenqi Hu, Xiyao Yang, Li Zhang, Min Yang, Shuang Cao

**Affiliations:** 1 The Second Department of Critical Care Medicine, The Second Affiliated Hospital of Anhui Medical University, Hefei, China; 2 Department of Nursing, The Second Affiliated Hospital of Anhui Medical University, Hefei, China

**Keywords:** alarm fatigue, intensive care unit, psychometrics, questionnaire, reliability, validity

## Abstract

**Introduction:**

This study aimed to translate the English version of the Charité Alarm Fatigue Questionnaire (not the original German version) into Chinese and evaluate its psychometric properties among ICU healthcare professionals in China.

**Methods:**

The Charité Alarm Fatigue Questionnaire was translated into Chinese following Brislin’s translation model and finalized after a pre-survey. A total of 674 questionnaires were distributed. The 634 valid responses were randomly split into two subsamples for exploratory factor analysis (*n* = 317) and confirmatory factor analysis (*n* = 317). Item analysis was performed using the critical ratio method, correlation coefficient method, and homogeneity test. Validity was assessed through content validity, construct validity, convergent validity, discriminant validity, and criterion-related validity. Reliability was evaluated using Cronbach’s *α* coefficient, McDonald’s *ω* coefficient, split-half reliability, and test–retest reliability.

**Results:**

The Chinese version consisted of 9 items and 2 dimensions, with a cumulative variance contribution rate of 68.346%. The item-level content validity index ranged from 0.80 to 1.00, and the scale-level content validity index/average was 0.954. The average variance extracted and composite reliability values for each dimension met the criteria, demonstrating good convergent and discriminant validity. The criterion-related validity was 0.567. Cronbach’s *α* coefficient was 0.855, McDonald’s *ω* coefficient was 0.842, split-half reliability was 0.871, and the test–retest reliability was 0.719. No significant floor or ceiling effects were observed.

**Discussion:**

The Chinese version demonstrates good psychometric properties and can be considered a valid tool for assessing alarm fatigue levels in China.

## 1 Introduction

Intensive Care Unit (ICU) is one of the most densely equipped and frequently alarmed departments in hospitals (Lewandowska et al., 2020). Alarm functions of various life support and monitoring devices —such as ventilators, cardiac monitors, and syringe pumps— are designed to promptly alert ICU healthcare professionals to changes in patients’ conditions and enhance patient safety (Ali et al., 2023). However, there are so many alarms in the ICU today that healthcare staff cannot respond to every alarm (Poncette et al., 2021). Studies have reported that each ICU bed triggers 100 to 400 alarms per day (Lewandowska et al., 2020; Tanner, 2013), of which approximately 80 to 99% are triggered incorrectly due to human, organizational, and technical factors (Bach et al., 2018; Seifert et al., 2021). ICU healthcare professionals spend nearly one-third of their working time managing false alarms (Lewandowska et al., 2020). Prolonged exposure to high-frequency and inefficient alarms can be hazardous and create alarm fatigue (Wilken et al., 2017).

Alarm fatigue refers to a condition in which ICU healthcare professionals become desensitized to alarms, leading to a diminished ability to appropriately respond to alarm-related procedures (e.g., delayed responses, turning down the alarm volumes, or turning off the alarms) (Wunderlich et al., 2023). This desensitization can compromise clinical care and increase the risk of adverse medical events (Bach et al., 2018; Rypicz et al., 2024; Gülşen and Arslan, 2025). According to the U. S. Food and Drug Administration (FDA) database and the Joint Commission’s Sentinel Event Database, 566 alarm-related deaths attributed to alarm fatigue occurred between 2005 and 2010, and 80 between 2009 and 2012 (Joint Commission, 2013). As reporting to the database is voluntary, this figure may be a conservative estimate (Rypicz et al., 2024; AAMI, 2011). The Association for the Advancement of Medical Instrumentation (AAMI) has indicated that the actual mortality rate associated with clinical alarms may be up to 10 times higher than the publicly reported rate (AAMI, 2011). In China, data from the national medical device adverse event monitoring system similarly indicate that alarm fatigue is a major cause of adverse events related to monitoring devices (Tian et al., 2022). Given the severity of the alarm problem, the scientific management of clinical alarms has attracted widespread attention. In 2020, the Health Technology Hazards Executive Brief listed alarms and alert overload as one of the top 10 health technology hazards (ECRI, 2020). Similarly, the China Hospital Association included “strengthening the security management of medical equipment and information systems” among the top 10 patient safety goals (China Hospital Association, 2017), and the issue of alarm management has been on the list for four consecutive years through 2020.

Alarm fatigue has become an increasing concern in contemporary clinical practice (Rypicz et al., 2024; Nyarko et al., 2023; Ruppel et al., 2023). As a latent psychological construct, it cannot be measured directly. Therefore, questionnaires are important tools to evaluate it (Rypicz et al., 2023). Several instruments have been developed to evaluate alarm fatigue among ICU healthcare professionals, including the Alarm Fatigue Scale (AFS) (Cho et al., 2016), the Nurses’ Alarm Fatigue Questionnaire (NAFQ) (Torabizadeh et al., 2017), and the Charité Alarm Fatigue Questionnaire (CAFQa) (Wunderlich et al., 2023). The Chinese version is hereafter referred to as C-CAFQa. The AFS developed by Cho et al. (2016) was based on seven items selected from previous studies conducted by Japanese Society for Occupational Health, Working Group for Occupational Fatigue (2002) and Kim and Sung (1998) without following a systematic scale development process. According to Boateng et al. (2018), scale development should begin with an initial pool approximately twice the size of the final scale, followed by refinement using theory-driven and statistical methods to ensure comprehensive construct coverage. However, these steps were not undertaken during the development of the AFS. Moreover, no subsequent psychometric evaluation—such as reliability or validity testing—was conducted to establish the instrument’s measurement properties. The NAFQ, developed by Torabizadeh et al. (2017), followed a relatively rigorous development process and demonstrated good reliability and validity. Although originally designed specifically for nurses, clinical alarm systems are complex, and excessive alarms result from multiple interacting factors; therefore, both nurses and physicians should have the opportunity to express their perspectives (Hüske-Kraus et al., 2018). While the NAFQ has been used with physicians in some studies (Bourji et al., 2020), its application to other healthcare professionals should be approached cautiously without further validation. To address the shortcomings of previous tools, Wunderlich et al. (2023) developed the CAFQa in 2023, which was specifically designed for ICU healthcare professionals. It contains 9 items that cover the dimensions of alarm stress and alarm coping, with a solid theoretical foundation and structural validity.

Currently, there is no standardized assessment tool for alarm fatigue among ICU healthcare professionals in China. The direct use of the English questionnaire may limit its applicability and measurement accuracy due to language differences, cultural variations, and different clinical practice environments (Beaton et al., 2000; Gjersing et al., 2010). Therefore, this study translated the English version of the CAFQa and conducted a psychometric evaluation to provide a valid and reliable tool for assessing alarm fatigue in Chinese ICUs. This study not only filled the gap of assessment tools in China but also provided theoretical support and practical reference for clinical managers to identify high-risk personnel, optimize the alarm systems, and enhance patient safety. To our knowledge, this represents the first cross-cultural adaptation of the CAFQa, thereby broadening the questionnaire’s international applicability.

## 2 Materials and methods

### 2.1 Study design and study population

A cross-sectional survey design was used in this study. Between January and April 2025, ICU healthcare professionals were recruited using convenience sampling from five randomly selected tertiary hospitals in Anhui Province. The inclusion criteria were: obtaining a practicing certificate; working in the ICU for≥6 months; and informed consent and voluntary participation. Exclusion criteria were: training or advanced training; absence from work during the survey period due to illness, maternity leave, or personal leave; and incomplete questionnaires.

According to Kendall’s recommendation (Zheng et al., 2023), the sample size for exploratory factor analysis (EFA) should be 5 to 10 times the number of items. For confirmatory factor analysis (CFA), a minimum of 200 participants is required (Kline, 2023). Considering an estimated 20% invalid response rate, a total of 674 questionnaires were distributed. After excluding 40 individuals who had worked in the ICU for less than 6 months, 634 participants were included in the final analysis, with an effective response rate of 94.07%. Among them, 153 were physicians, and 481 were nurses. The average age was 32.57 years (range 22–60 years). There were 201 males and 433 females. The sociodemographic and clinical characteristics of the participants were detailed in Table 1.

**Table 1:** Sociodemographic and clinical characteristics of participants (*N* = 634).

Variable	Mean ± SD or *n* (%)
Age (years)	32.57 ± 6.32
Gender	
Male	201 (31.7)
Female	433 (68.3)
Profession	
Physician	153 (24.1)
Nurse	481 (75.9)
Education	
College or bachelor	566 (89.3)
Master	59 (9.3)
Doctor	9 (1.4)
ICU type	
Integrated ICU	521 (82.2)
Internal medicine ICU	58 (9.1)
Surgical ICU	32 (5.0)
Others	23 (3.6)
ICU years of experience	
<1 year	51 (8.0)
1 ≤ 5 years	180 (28.4)
5 ≤ 10 years	181 (28.5)
10 ≤ 15 years	155 (24.4)
≥15 years	67 (10.6)
Frequency of night shifts	
No night shifts	48 (7.6)
Once a week	106 (16.7)
2 times a week	287 (45.3)
3 times a week	160 (25.2)
4 times a week or more	33 (5.2)

ICU, Intensive Care Unit; SD, standard deviation.

### 2.2 Ethical considerations

This study was approved by the Ethics Committee of the Second Affiliated Hospital of Anhui Medical University (YX2025–096). All participants gave informed consent and voluntarily participated in this study.

### 2.3 Translation and cross-cultural adaptation

With permission from the original authors, the CAFQa was translated into Chinese following Brislin’s translation model (Jones et al., 2001). The process is as follows: (1) Forward Translation: Two independent translators—a medical doctor (IELTS 7.0, 6 years of study in the United States) and a nursing Ph. D. (15 years of clinical experience in the U. S.)—each translated the English version into Chinese (versions T1 and T2). Discrepancies were resolved through discussion by the research team, and a unified preliminary version (Version A) was developed (2) Back Translation: Two medical doctors who had no prior exposure to the original scale and were proficient in Chinese and English (each with three years of study in the United States) independently back-translated version A into English (versions T3 and T4). These versions were then compared, discussed, and revised by the research team to form a unified back-translated version (Version B). (3) Cross-Cultural Adaptation: A panel of five experts, —two ICU medical professors, one ICU nursing professor, one public health professor, and one psychology professor (all holding doctoral degrees and over 20 years of professional experience)—evaluated Versions A and B, along with the original English version, based on the cross-cultural adaptation guidelines proposed by Guillemin et al. (1993). Linguistic accuracy and cultural relevance were assessed, resulting in the finalized Chinese version (Version C). All experts agreed that no cultural modifications were required.

### 2.4 Pre-survey

A pre-survey was conducted to assess the feasibility and comprehensibility of the questionnaire. Based on Johanson and Brooks’s (2009) recommendation, a sample size of 24–36 is appropriate. Accordingly, 30 ICU healthcare professionals were recruited. They completed the questionnaire independently and evaluated its format, content, clarity, readability, and response options. All reported that the scale was easy to understand, with no ambiguities or suggestions for revision. Completion time was 3 to 5 min.

### 2.5 Measurement instruments

#### 2.5.1 General information questionnaire

This included age, gender, profession, education, ICU type, ICU years of experience, and frequency of night shifts.

#### 2.5.2 Charité Alarm Fatigue Questionnaire (CAFQa)

Professor Poncette et al. (2021) developed the CAFQa in 2023 to assess alarm fatigue among ICU healthcare professionals (Wunderlich et al., 2023). It consisted of two dimensions and nine items, rated on a 5-point Likert scale ranging from 0 (“I do not agree at all”) to 4 (“I very much agree”). Items 3, 4, 5, and 7 were reverse-scored. The total score ranged from 0 to 27, with higher scores indicating more severe alarm fatigue.

#### 2.5.3 Nurse alarm fatigue scale (NAFS)

NAFS (Torabizadeh et al., 2017) was developed to evaluate alarm fatigue among ICU nurses. It contained 13 items describing different aspects of alarm fatigue. The scale used a 5-point Likert scale ranging from 0 (“never”) to 4 (“always”), with items 1 and 9 being reverse-scored. The total score ranged from 0 to 52, with higher scores indicating higher alarm fatigue. The Cronbach’s *α* coefficient was 0.91 for the original scale (Torabizadeh et al., 2017) and 0.77 for the Chinese version (Jie et al., 2021).

### 2.6 Data collection

Researchers explained the purpose of the study to the participants, and the paper questionnaires were distributed only after obtaining consent. All questionnaires were distributed and collected on-site. Researchers used standardized instructions to explain items and filling methods. After completion, the questionnaires were promptly collected, reviewed, and invalid responses were excluded. Completion time ranged from 5 to 10 min.

### 2.7 Statistical analysis

Data were entered using EpiData 3.1 and analyzed with SPSS 26.0 (IBM Corp., Armonk, NY) and AMOS 24.0 (IBM Corp). Descriptive statistics were used to summarize participant characteristics. *p* < 0.05 was statistically significant.

#### 2.7.1 Item analysis

Critical Ratio (CR) Method: Independent samples t-tests were conducted to compare participants scoring in the upper 27th percentile with those in the lower 27th percentile of total scale scores. Items with CR values <3 or *p* > 0.05 were excluded (Zheng et al., 2023). Correlation Coefficient Method: The correlation coefficient (*r*) was calculated between each item and the total scale score. Items with *r* < 0.4 or *p* > 0.05 were excluded (Zheng et al., 2023). Homogeneity test (Cronbach’s *α* Coefficient): Items were excluded if their removal increased the overall Cronbach’s α coefficient (Zheng et al., 2023). Floor and ceiling effects of the Chinese version of the CAFQa (C-CAFQa) were evaluated, and the floor or ceiling effect was considered present if more than 15% of participants achieved the lowest or highest possible score (Terwee et al., 2007).

#### 2.7.2 Validity analysis

##### 2.7.2.1 Content validity

Five experts were invited to assess the content validity. They evaluated each item using a 4-point Likert scale (1 = “not relevant” to 4 = “very relevant”). The item-level content validity index (I-CVI) was calculated by dividing the number of experts scoring an item as 3 or 4 by the total number of experts. The scale-level Content Validity Index/Average (S-CVI/Ave) was computed as the mean of all I-CVIs. Content validity was considered acceptable if I-CVI ≥ 0.78 (Polit and Beck, 2006), and S-CVI/Ave ≥ 0.90 (Polit et al., 2007).

##### 2.7.2.2 Construct validity

The 634 participants were randomly divided into two equal groups (*n* = 317 each) for EFA and CFA using computer-generated randomization. Sampling adequacy was examined using the Kaiser-Meyer-Olkin (KMO) and Bartlett’s test of sphericity. According to commonly accepted criteria, a KMO value above 0.60 indicates acceptable sampling adequacy for factor analysis, and Bartlett’s test should be statistically significant (*p* < 0.05) (Schreiber, 2021). Principal axis factoring (PAF) with Promax oblique rotation was used to extract factors with eigenvalues >1.0 and factor loadings >0.40 (Bibi et al., 2023). Subsequently, CFA was conducted using maximum likelihood estimation to confirm the identified factor structure.

##### 2.7.2.3 Convergent and discriminant validity

If the average variance extracted (AVE) > 0.5 and composite reliability (CR) > 0.6, it was considered acceptable (Bibi et al., 2023); if the $\sqrt{\mathrm{AVE}}$ of a variable was greater than the correlation coefficient between the variable and all other variables, it suggested good discriminant validity (Cheung et al., 2024).

##### 2.7.2.4 Criterion-related validity

The Pearson correlation coefficient (*r*) indicated the degree of correlation between the C-CAFQa and the Chinese version of NAFS. According to established criteria, r ≥ 0.30 indicates moderate and r ≥ 0.50 indicates strong criterion-related validity (Cohen, 1988).

#### 2.7.3 Reliability

Internal consistency reliability was assessed using Cronbach’s *α* (> 0.7) (Dunn et al., 2014), McDonald’s *ω* (> 0.8) (Dunn et al., 2014; Taylor, 2021), and split-half reliability (> 0.8) (Koo and Li, 2016). External consistency was evaluated by retesting 30 randomly selected participants after 2 weeks, with a reliability threshold of > 0.7 (Taylor, 2021).

## 3 Results

### 3.1 Item analysis

As shown in Table 2, CR values ranged from 16.50 to 23.29 (all >3.0 and *p* < 0.01), while the r values ranged from 0.606 to 0.721 (all >0.4 and *p* < 0.01). Additionally, removing any item did not increase Cronbach’s *α* coefficient. Therefore, all 9 items were retained. The floor effect was 4.10% and the ceiling effect was 0.47%, below 15%, indicating no significant floor or ceiling effects.

**Table 2:** Item analysis of the C-CAFQa (*N* = 634).

Items	CR	ITC	Cronbach’s α if item deleted
Factor 1			
N1	19.18**	0.702**	0.837
N2	21.81**	0.713**	0.836
N6	22.40**	0.689**	0.839
N8	23.29**	0.706**	0.837
N9	20.12**	0.721**	0.835
Factor 2			
N3	20.93**	0.672**	0.841
N4	16.50**	0.606**	0.849
N5	20.00**	0.651**	0.844
N7	21.31**	0.666**	0.842
Total			0.855

C-CAFQa, Chinese version of Charité Alarm Fatigue Questionnaire; CR, critical ratio (T); ITC, item-total correlation; **p < 0.01.

### 3.2 Validity

#### 3.2.1 Content validity

The I-CVI was 0.80–1.00 (>0.78), and the S-CVI/Ave was 0.954 (>0.9), meeting the acceptance criteria.

#### 3.2.2 Construct validity

**Figure 1:**
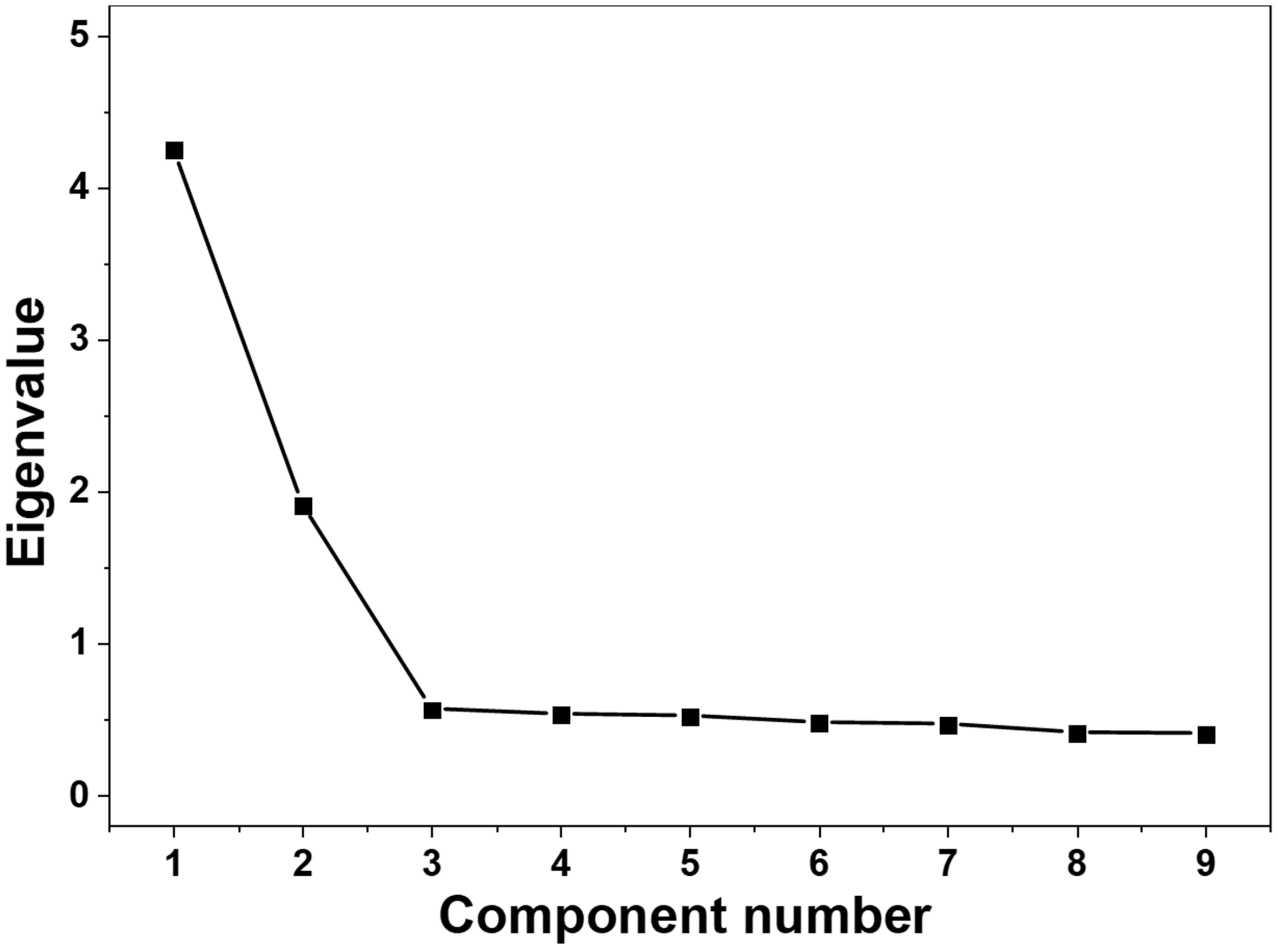
Scree plot. The scree plot shows a clear inflection after the second point. Consistent with the Kaiser criterion (eigenvalues > 1), two factors were retained for further analysis.

The EFA results showed that Bartlett’s test of sphericity was significant (*χ*^2^ = 1335.789, df = 36, *p* < 0.001) and the KMO was 0.896, indicating good sampling adequacy for factor analysis. Two common factors with eigenvalues greater than 1 were extracted, consistent with the original questionnaire structure, contributing 68.346% of the cumulative variance (Table 3). Similarly, the scree plot (Figure 1) showed a clear inflection after the second point, indicating that only the first two points had eigenvalues greater than 1, which supports the retention of a two-factor structure. Factor loadings were all >0.4, and no items were deleted (Table 3).

**Table 3:** Exploratory factor analysis and convergent validity of the C-CAFQa (*N* = 317).

Items	Factor loading		AVE	CR (Composite Reliability)
	1	2		
Factor 1: Alarm stress			0.611	0.887
N1: With too many alarms on my ward, my work performance and motivation decreases	**0.772**	0.323		
N2: Too many alarms trigger physical symptoms for me, e.g., nervousness, headaches, sleep disturbances	**0.778**	0.312		
N6: Alarms reduce my concentration and attention	**0.723**	0.313		
N8: My or neighboring patients’ alarms or crisis alarms frequently interrupt my workflow	**0.797**	0.347		
N9: There are situations when alarms confuse me	**0.772**	0.379		
Factor 2: alarm coping			0.611	0.862
N3: In my ward, a procedural instruction on how to deal with alarms is regularly updated and shared with all staff	0.377	**0.772**		
N4: Responsible personnel respond quickly and appropriately to alarms	0.305	**0.746**		
N5: The audible and visual monitor alarms used on my ward floor and cockpit allow me to clearly assign patient, unit, and urgency	0.342	**0.779**		
N7: Alarm limits are regularly adjusted based on patients’ clinical symptoms (e.g., blood pressure limits for condition after bypass surgery)	0.321	**0.788**		
Eigenvalue	4.272	1.879		
variance contribution, %	47.465	20.881		
Cumulative variance contribution, %		68.346		

C-CAFQa, Chinese version of Charité Alarm Fatigue Questionnaire; AVE, average variance extracted values. Bold values indicate primary factor loadings (≥0.40), while non-bold values represent cross-loadings.

**Figure 2:**
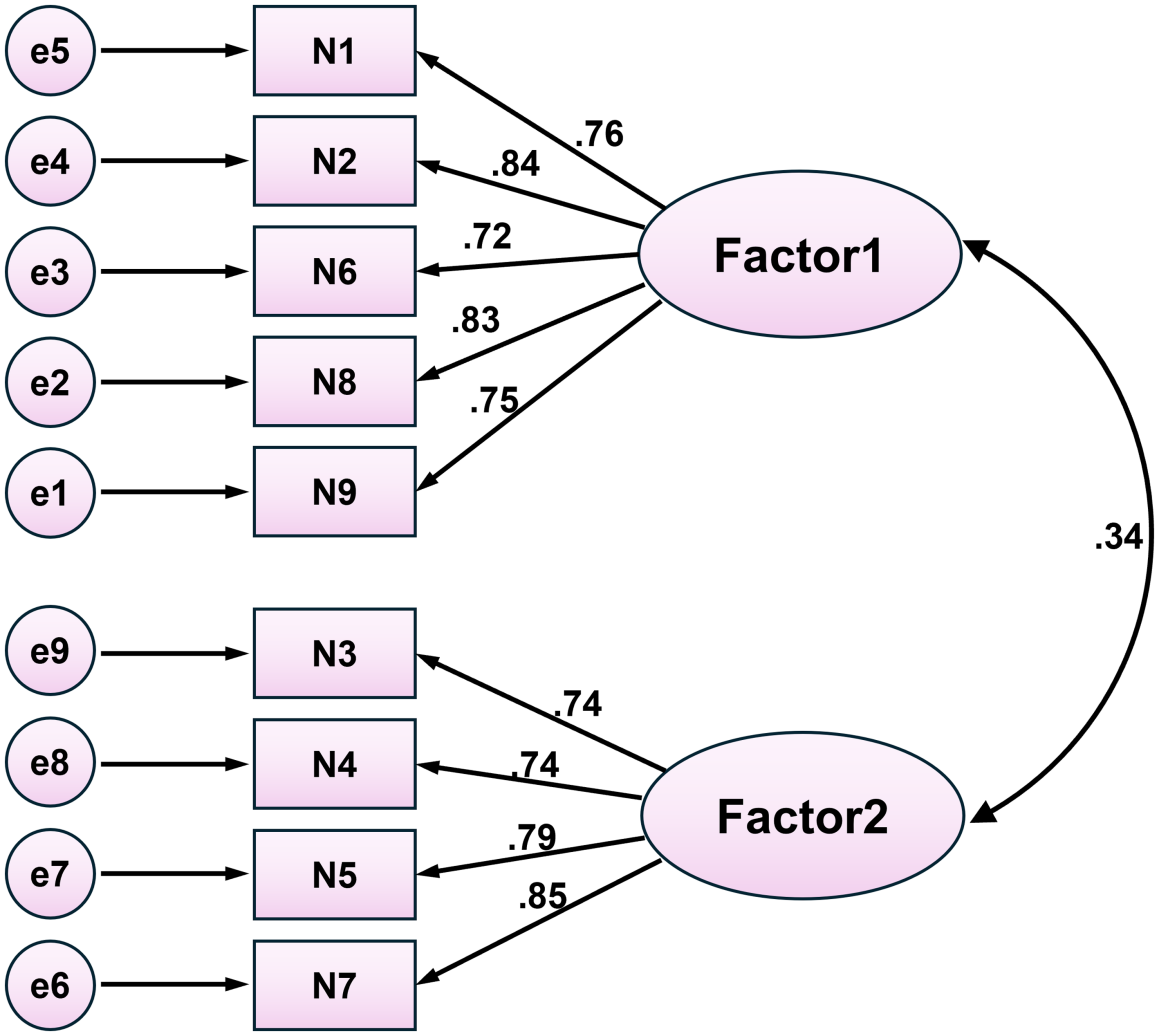
Confirmatory factor analysis. All factor loadings are standardized, all *p* < 0.001; Factor 1: Alarm stress; Factor 2: Alarm coping.

Table 4 showed the model fit indices and evaluation criteria, demonstrating that the model fits the data well. In the CFA, the two dimensions were treated as latent variables, and the nine items were used as observed variables to construct the model. The results of the CFA are shown in Figure 2, with factor loadings ranging from 0.72 to 0.85.

**Table 4:** Goodness-of-fit indices of the C-CAFQa (*N* = 317).

Fit indices	*χ*^2^/DF	RMSEA	GFI	CFI	IFI	TLI
Acceptable value	<3	<0.08	≥0.9	≥0.9	≥0.9	≥0.9
Observed value	2.600	0.071	0.956	0.971	0.971	0.960

C-CAFQa, Chinese version of Charité Alarm Fatigue Questionnaire; *χ*^2^/DF, chi-square/degree of freedom; RMSEA, root mean square error of approximation; GFI, goodness of fit index; CFI, comparative fit index; IFI, incremental fit index; TLI, Tucker–Lewis index.

#### 3.2.3 Convergent and discriminant validity

As shown in Table 3, all AVE values were >0.5 and all CR values were>0.6. Additionally, the $\sqrt{\mathrm{AVE}}$ values (Factor 1:0.7814, Factor 2:0.7813) were all greater than the correlation coefficients (0.269).

#### 3.2.4 Criterion-related validity

Pearson’s correlation coefficient between the C-CAFQa and NAFS total scores was statistically significant (r = 0.567, p < 0.001).

### 3.3 Reliability

Cronbach’s *α* for the total scale was 0.855 (95% CI: [0.834, 0.873]), McDonald’s *ω* was 0.842 (95% CI: [0.816, 0.864]), split-half reliability was 0.871, and test–retest reliability was 0.719 (95% CI: [0.501, 0.858]). For the two dimensions, Cronbach’s α were 0.880 and 0.857, McDonald’s ω were 0.881 and 0.857, and split-half reliability were 0.899 and 0.837.

## 4 Discussion

Adverse events caused by alarm fatigue are common and frequently underestimated (Rypicz et al., 2024; AAMI, 2011). It has become a critical issue that cannot be ignored (Ruppel et al., 2023). In China, research on alarm fatigue remains in its early stages, particularly due to the lack of standardized assessment tools for ICU healthcare professionals. Moreover, most existing studies have focused on nurses (Storm and Chen, 2021; Salameh et al., 2024; Lu et al., 2024), with less attention given to physicians who also experience alarm fatigue (Bourji et al., 2020; Cole et al., 2024). To address this gap, this study introduced the CAFQa, providing a standardized tool for clinical assessment and management.

Following COSMIN recommendations for cross-cultural adaptation and measurement property evaluation, this study used the Brislin translation model, expert consultation, and cultural adaptation to ensure semantic, conceptual, and cultural equivalence of the questionnaire. This process preserved the theoretical integrity of the original version while aligning with Chinese linguistic and cultural norms (Çapik et al., 2018). Item analysis, validity, and reliability testing supported the retention of all nine original items, and the factor structure remained consistent with the original scale.

Item analysis, a key step in scale refinement, facilitated the evaluation of item discrimination and internal consistency (Streiner et al., 2016; Kyriazos and Stalikas, 2018). In this study, all items met the retention criteria based on the critical ratio method, correlation coefficient method, and homogeneity test, with the C-CAFQa demonstrating good item discrimination.

Although alarm fatigue is a global issue, its manifestations may vary due to cultural and regulatory differences. For example, the high-intensity work and centralized alarm systems in Chinese ICUs may exacerbate fatigue among healthcare professionals. Therefore, the instrument must be revalidated in the target population after translation (Cha et al., 2007). Validity refers to the extent to which an instrument accurately measures its intended construct (Ahmed and Ishtiaq, 2021). In this study, content validity, construct validity, convergent validity, discriminant validity, and criterion-related validity were assessed. For content validity, both I-CVI and S-CVI/Ave exceeded the recommended thresholds, indicating adequate content representation (Polit and Beck, 2006; Polit et al., 2007). EFA identified two factors—alarm stress and alarm coping—explaining 68.346% of the total variance, consistent with the original version and supporting cross-cultural structural stability (Wunderlich et al., 2023; Wunderlich et al., 2024). The cross-cultural consistency may result from the original authors’ neutral item descriptions, enabling broader applicability in diverse ICU settings. Factor 1 (alarm stress) reflects the physical and psychological burden of frequent alarms, while the alarm coping factor refers to the behavioral strategies to manage alarm fatigue. This two-factor structure aligns with occupational stress and coping theories (Folkman and Moskowitz, 2004) and prior studies on alarm fatigue (Cvach, 2012). However, factor retention based on eigenvalues and scree plot inspection may be subjective and prone to over-extraction; future studies should apply more robust methods, such as parallel analysis or the minimum average partial (MAP) test, to further strengthen construct validity. Furthermore, CFA also demonstrated that the model was well-fitted (Zhang, 2017). Convergent validity was assessed using classical statistical methods (AVE and CR) in structural equation modeling, and the results indicated good convergent validity (Cheung et al., 2024). Unlike the original scale, which relied on participants’ subjective estimates of alarm fatigue and false alarm rates (Wunderlich et al., 2024), this study employed standardized and objective psychometric methods consistent with COSMIN recommendations, enhancing the transparency and replicability of the validation. In addition, the √AVE value was greater than the correlation coefficient, further indicating that the scale had good discriminant validity (Cheung et al., 2024). The correlation coefficient between the C-CAFQa and the Chinese version of NAFS was 0.567, demonstrating acceptable criterion-related validity (Cheung et al., 2024). Overall, the C-CAFQa had good validity.

To comprehensively assess the internal consistency and temporal stability of the questionnaire (Koo and Li, 2016), this study employed Cronbach’s *α*, McDonald’s *ω*, split-half reliability, and test–retest reliability (Revelle and Condon, 2019). The results indicated good internal consistency and acceptable but moderate temporal stability. The test–retest reliability should be interpreted cautiously, as changes in alarm exposure and work environment during the two-week interval may influence participants’ responses. Compared with the original version, the Chinese version showed higher reliability, particularly in the second factor. This difference may reflect clearer translation, improved contextual relevance in Chinese ICUs, or cultural variations in alarm fatigue perception; however, these explanations remain speculative without direct empirical comparison.

This study not only validated the psychometric properties of the CAFQa but also provided a practical tool for clinical practice in Chinese ICUs. The C-CAFQa can be used for routine assessment of healthcare professionals’ alarm fatigue levels, particularly through the two subscales of “alarm stress” and “alarm coping,” which help identify high-risk populations and potential intervention priorities, such as excessive exposure to alarm stimulation or lack of effective coping strategies. This tool can also serve as a quantitative indicator for evaluating the effectiveness of alarm intervention measures, providing evidence to optimize ICU alarm management, improve the work environment, and enhance patient safety.

Despite the rigorous cross-cultural translation process and psychometric evaluations conducted in this study, there were some limitations. First, the translation was based on the English version of the CAFQa provided by the original authors rather than the original German version, which may have introduced semantic drift and limited strict measurement equivalence and cross-cultural comparability. Future studies should consider direct translation from the original version. Second, content validity was assessed by a small expert panel with limited disciplinary diversity; the absence of biomedical engineers and patient safety specialists may have restricted the breadth of perspectives. Future studies should include a larger, interdisciplinary expert panel to strengthen content validity. Third, criterion-related validity was assessed using only a self-report measure (NAFS), which may introduce common method bias and limit evidence to self-reported constructs; future studies should validate the scale against objective, behavioral, or clinical criteria. Fourth, the two-week test–retest interval may be insufficient to assess temporal stability while potentially being affected by intervening experiences; future studies should consider longer intervals with documentation of interim alarm exposure. Lastly, this study employed convenience sampling and was conducted in hospitals within a single province, which may limit representativeness and introduce potential selection bias. Regional differences in healthcare systems, service delivery models, and patient characteristics across China may influence alarm fatigue levels. Therefore, future multicenter studies across diverse regions are needed to further validate the national applicability and generalizability of the scale.

## 5 Conclusion

This study translated and validated the CAFQa into Chinese. The Chinese version demonstrated good reliability and validity and can be used to assess alarm fatigue among ICU healthcare professionals in China. Its application may help identify alarm fatigue levels, guide targeted interventions, and improve patient safety and care quality in ICU settings.

## Data Availability

The raw data supporting the conclusions of this article will be made available by the authors, without undue reservation.
